# Challenging Reward Structures and Organizational Cultures that Propagate Stem Cell Hyperbole

**DOI:** 10.1007/s12015-025-10955-z

**Published:** 2025-08-18

**Authors:** Annie Trinh, Leigh Turner

**Affiliations:** 1 Department of Microbiology & Molecular Genetics, University of California-Irvine, Irvine, CA 92697 USA; 2 NSF-Simons Center for Multi-scale Cell Fate Research, University of California-Irvine, Irvine, CA 92697 USA; 3 Edwards Lifesciences Cardiovascular Innovation & Research Center, University of California-Irvine, Irvine, CA 92697 USA; 4 Department of Health, Society, and Behavior, Joe C. Wen School of Population and Public Health, University of California-Irvine, Irvine, CA 92697 USA; 5 Center for Health Ethics, University of California-Irvine, Irvine, CA 92697 USA; 6 Sue and Bill Gross Stem Cell Research Center, University of California-Irvine, Irvine, CA 92697 USA

**Keywords:** Responsible science communication, Stem cell hyperbole

## Abstract

How science is communicated shapes public understanding of science and informs decision-making by patients, research participants, policymakers, public funding agencies, private philanthropic organizations, and corporations. Responsible science communication is a collective responsibility of scientists. Accurate reporting is also a crucial feature of news media coverage of scientific research. Unfortunately, scientists, journalists, and other parties sometimes make hyperbolic claims that go beyond available evidence and exaggerate the significance of particular research findings. This phenomenon is evident in the rapidly evolving and highly competitive fields of stem cell biology and regenerative medicine, though hyperbolic representations have also been documented in such fields as artificial intelligence, genomics, precision medicine, and synthetic biology. Stem cell hyperbole is shaped and promoted by systemic factors. We highlight the continued significance of responsible communication of stem cell science across news media and social media, especially in an era where there are powerful incentives to make hyperbolic claims. While such norms as truthfulness, accuracy, and accountability might seem self-evident, contemporary incentive structures and organizational cultures play an important role in promoting hyperbolic representations and other inaccurate representations of scientific research. Finally, we propose recommendations for supporting and sustaining research cultures that prioritize honesty and accuracy in science communication and public engagement.

## Introduction

How science is communicated by researchers and research institutions informs public understanding and shapes the expectations of scientists and nonscientists alike [1]. Science communication involves far more than merely transmitting research findings to the scientific community and “the public.” Science communication informs decision-making by patients and patient organizations, shapes understanding of health researchers and research participants, and influences priority-setting and resource allocation decisions by public funding agencies and companies in the private sector. How science is framed, described, interpreted, and put in context affects health and science policies and policy-making processes, public debates about different fields of scientific research, and public understanding of a range of related ethical, legal, and social concerns [2–4]. Responsible science communication is important across scientific fields, though here we examine its particular significance for public understanding of stem cell biology and regenerative medicine. In recent years, these fields have experienced notable growth with research focused on stem cells growing approximately twice as fast as the world average rate of all research in 2013 [5]. Yet, studies document that few stem cell-based therapies have made it to routine clinical use, and many stem cell-based medical products still lack standardization and convincing evidence of safety and efficacy [6, 7]. These rapidly evolving research fields also experience considerable funding unpredictability, particularly especially at the federal level, and are therefore highly competitive [8, 9].

Researchers studying patterns in news media coverage of stem cell research and regenerative medicine have documented that news outlets often highlight the therapeutic promise of stem cells in treating illnesses and injuries, and emphasize the clinical potential of translational stem cell research [10, 11]. This narrative framing of news coverage of stem cell research is understandable given how many basic science, pre-clinical, and clinical studies investigate the use of stem cell interventions in addressing a wide range of diseases and injuries. One of the main justifications advocates use to advance the case for funding stem cell research is that the field has significant potential to transform the treatment of a multitude of illnesses and injuries. Such news coverage might also reflect a more general pattern of depicting scientific research in positive and optimistic terms. Research institutions, academic journals, and scientists are active participants in this process of news production, and are typically eager to have their studies publicized in favorable terms. They use press releases, news media alerts, social media platforms, and other tools to bring such studies to the attention of journalists and the public. [4, 12–14].

There is nothing intrinsically concerning about news media outlets describing the potential clinical significance of particular research studies if news coverage is accurate, provides realistic timelines for development, and does not assume that early-stage investigational products will inevitably become treatments or even outright cures. Given the important place of scientific research in society, news coverage of scientific findings is vital for public understanding and debate [2, 15–17]. However, news media reports become problematic when they contain exaggerated, unwarranted representations. While optimistic and enthusiastic framings of stem cell research might be welcomed by stem cell researchers trying to secure research funding in highly competitive environments, hyperbolic news media coverage can have detrimental effects. For example, acknowledging the many factors that influence whether citizens find scientists and science trustworthy and credible, the relationship between scientists and the public is made vulnerable when science is misrepresented during communication [3, 10, 18–20]. Such misrepresentations can generate concerns about the trustworthiness, integrity, and credibility of scientists, their research, and the institutions where they work.

“Science hype” is a contested and complex phenomenon, criticized by many scholars for its misrepresentations but also framed in more positive terms by some researchers that see promissory and anticipatory rhetoric about future scientific breakthroughs as a necessary means to generate public support and funding for emerging fields of study. In general, science hype refers to exaggerated, overly optimistic representations concerning the current state or anticipated future benefits of particular domains of scientific research [3, 11, 21]. Hyperbolic claims are typically boosterish in nature, though researchers have also explored forms of hyperbolic rhetoric tied to exaggerated depictions of unwanted outcomes and potential misuses and ethical abuses of particular research findings and technologies [22].

Science hyperbole can take various forms and should be considered as a continuum or range of claims from comparatively minor exaggerations to more significant and consequential misrepresentations. While some supporters of boosterish rhetoric may assert that minor exaggerations of scientific findings may be helpful if they evoke positive responses from the public toward scientific research or persuade younger individuals to engage in scientific research, science hyperbole, including stem cell hype, can also provide a fertile social environment for more serious forms of misrepresentation such as the deceptive advertising and outright fraud used by some businesses engaged in direct-to-consumer marketing of unproven and unapproved stem cell interventions and other purported regenerative medicine products. In recent years, researchers have described hyperbolic claims becoming increasingly prevalent in the fields of stem cell biology and regenerative medicine [10, 11, 21, 23].

In an era of digital communications where information, whether evidence-based or unsupported by credible evidence, spreads widely and with great speed [14, 24–26], it is crucial to address stem cell hyperbole and to develop effective strategies for supporting responsible, evidence-based science communication. We examine important drivers of science hype and consider what roles scientists and journalists should play in promoting ethical and responsible science communication. We also identify contemporary challenges related to using social media platforms as sites for accountable science communication and public engagement. We highlight the importance of developing, supporting, and incentivizing research environments that value and reward honesty, transparency, and caution in claims-making while enabling scientists and journalists to fulfill their crucial roles in promoting responsible communication of science. Fostering such research environments and organizational cultures will not guarantee that stem cell hyperbole will disappear. However, they can create institutional spaces for more responsible and accountable forms of science communication.

## Stem Cell “Hype”

“Promissory rhetoric” and anticipatory claims concerning the potential of particular scientific fields help “mobilize the scientific community and potential funders” [3] by making claims about the future health, economic, and other benefits that will flow from supporting research in specific domains. In areas where scientists operate in highly competitive environments, where many credible, even laudable scientific research studies are destined to go unfunded, it is unsurprising that hyperbolic claims are used to generate interest, attract financial support, and draw support for particular fields of study. However, promissory rhetoric about desired futures can shift easily from credible, appropriately cautious representations to concerning exaggerations.

In the fields of stem cell biology and regenerative medicine, in which funding is highly competitive and unpredictable, such hyperbole regarding the promise of stem cell research—as with science hype in such fields as artificial intelligence, genomics, precision medicine, and synthetic biology—can misinform and mislead patients, research participants, and other members of society. In some political contexts, stem cell hyperbole can be used strategically to influence public opinion and policymaking, especially in jurisdictions where the morality and legality of certain types of stem cell research are actively contested. In some cases, although hype may generate initial excitement toward supporting stem cell research, it can also promote unrealistic expectations that ultimately will not be met, which could compromise long-term support and public perceptions of the field [27]. While the relationship between science hype and public trust in science is complicated and multilayered, with some researchers arguing there is little empirical evidence to support the claim that hyperbole plays a causal role in compromising public trust in research, we share the concerns of other scholars that exaggerated claims can undermine the credibility and trustworthiness of those individuals and institutions responsible for making them [1, 3, 18–21, 28].

Some researchers highlight the role of journalists in offering hyperbolic accounts of scientific research, suggesting that lack of domain-specific expert knowledge, financial pressures news media organizations face to “sell news,” and other factors drive such misrepresentations. However, hype can arise from all stages of the complex processes from which news media reports emerge, and from different components of the complicated assemblies that contribute to the production of news media reports. All parties engaged in science communication should therefore conduct themselves responsibly [29, 30]. While there are examples of hyperbolic news media coverage of stem cell research, as with other fields of such, such restricted ways of considering the sources of science hype risk scapegoating journalists. They fail to acknowledge adequately the often active, participatory role of scientists and research institutions in the construction of hyperbolic accounts of stem cell research and other fields of study [31, 32]. Such reductionistic framings mischaracterize the organizational environments and reward structures from which hyperbolic claims about science emerge.

## Incentive and Disincentive Structures

As Timothy Caulfield emphasizes in several important articles on the subject, “science hype” is generated by many actors and systemic pressures and reward structures [3, 10, 21]. To sustain and advance their careers, scientists in many fields must secure research funding, publish their findings in a timely manner, and establish themselves as leaders in their field of study. For faculty members based at research universities, the ability to demonstrate a consistent, successful record of funding and a strong record of publishing peer-reviewed studies in high-impact journals is often a critical requirement for obtaining academic tenure. Given that not being awarded tenure and a permanent academic appointment can result in researchers having to leave their home institutions and possibly even exit their chosen field of study, the tenure review process is a powerful example of academia’s broader “publish or perish” culture [33].

In such highly competitive research environments, the professional success and reputation of scientists is typically defined in part by such metrics as total funding generated, impactful articles published, and number of articles published. Tenure files, applications for promotion, and merit review packages for academic researchers often list the precise impact factors of the journals in which such researchers publish, the exact number of articles they have published, and the total amount of internal and external funding they have generated within defined review periods. Failure to secure substantial external funding and achieve a high and sustained level of research productivity can result in denial of tenure decisions or other types of career setbacks such as being assigned smaller laboratory spaces or having teaching duties or clinical responsibilities increased [33, 34]. In the case of “soft money” positions in medical schools, academic health centers, and other fields in the health sciences, salaries of researchers often are linked to obtaining sufficient levels of external research funding because employers do not provide total salary compensation as a long-term “hard money,” guaranteed institutional commitment [35]. Researchers can have to generate anywhere from a modest percentage of their salary to their entire salary to receive full pay and remain in their positions [35, 36].

For stem cell research in particular, funding is extremely unpredictable and highly competitive [8, 9]. Such financial pressures, employment metrics, and audit cultures might seem distinct from the subject of science hyperbole, but such highly competitive research environments can create a climate in which making hyperbolic claims is rewarded and can even come to seem necessary as a matter of continued employment and professional survival. For example, researchers may intentionally or unintentionally engage in hyperbole to secure funding. However, this is not a sustainable approach to funding stem cell research long-term if the supply ultimately cannot meet the demand of the public (i.e., when lofty promises of future scientific breakthroughs generate comparatively modest results in the eyes of the public) [27].

Institutional expectations to obtain substantial research funding and publish peer reviewed scholarship at a frequent pace and in high impact factor journals can result in researchers experiencing pressures to not only display high levels of research productivity, but also to present their scientific work in ways that can at times be misleading. Such representations can be made in various contexts, such as communicating research findings to journalists, “pitching” research programs to potential philanthropic funders, submitting grant applications to highly competitive funding agencies, and interacting with investors. Certain “hyperbolic” phrases have become associated with high impact work in biomedical research. Phrases such as “cutting-edge,” “paradigm-shifting”, and “breakthroughs” are used to attract the attention of editors and reviewers at prestigious journals. Such promotional language has been observed to be more frequently used in recent grant applications and research papers [37–39]. In contrast, engaging in responsible science communication by, for example, outlining extended timelines accurately or highlighting negative study results, and contributing to substantial public outreach and community engagement often are less commonly rewarded by institutions. Many high-impact, high profile, news-shaping academic journals contribute to this phenomenon by playing their own important role in perpetuating a highly competitive research environment in which studies must be framed as novel, exciting, and “pathbreaking” to have any prospect of being published [40].

When responding to the structure of incentives and disincentives in such intensely competitive research environments, researchers sometimes turn to hyperbolic rhetoric and exaggerate the significance of their research. The use of hyperbole can lead to study results being misrepresented, and to the exaggeration of potential clinical and commercial applications of research findings [3, 18]. Researchers have found examples of hyperbole in scientific publications and in representations of scientific research to the wider community, such as in press releases regarding the significance, clinical translation, and potential commercialization of academic research findings [18].

## Changing Incentive Structures and Organizational Cultures to Support More Responsible Science Communication

Systemic pressures driving science hype have been examined in numerous publications [3, 10, 18, 21]. However, proceeding from documenting key drivers of science hype to promoting meaningful institutional reforms is a significant challenge because existing incentive structures and organizational cultures are well-established and resistant to significant change. The social structures, incentive schemes, and professional rewards comprising “publish or perish” academic culture are surprisingly resistant to reform even though significant problems with such competitive research contexts are well-documented. While some researchers are willing to transform the system and attempt to build different scientific cultures and organizational contexts, many scientists are “too busy just trying to survive” to be capable of working from within to change the entrenched system [41].

Sutter refers to existing recommendations from scientists to foster research integrity amidst science hype, such as the San Francisco Declaration on Research Assessment [42] and the Hong Kong Principles [43]. Sharing the common theme of reducing the significance of journal-based metrics such as impact factor, these recommendations identify other valuable standards for evaluating scientific output and merit including making data and methods publicly available and influencing policy and practice. Shifting measurement standards beyond amount of research funding generated, number of publications, and impact factors of journals to alternative frameworks for evaluation could help alleviate pressures associated with the highly competitive environment produced by the current “publish or perish” culture. Other alternative standards for evaluating scientific output and merit could include contributions to public resources for advancing scientific research, community outreach and public engagement, and increased transparency in reporting experimental design and data interpretation.

Reducing exaggerated representations of science will require developing and supporting an academic research culture that encourages and rewards academic honesty, accurate, and evidence-based representations of research findings, and scientific rigor, even if such changes reduce the number of publications researchers produce and extend the timeline needed to generate meaningful research findings and validate their significance. To increase responsible communication in stem cell research and the wider field of biomedical research, we must also confront and address the systemic pressures that exacerbate science hype and other types of misrepresentation such as “spinning” the results of particular studies (i.e., exaggerating scientific results and/or conclusions in a way that could distort audience interpretations) to make their findings seem more significant.

## Hyperbole, Science Journalism, and Cutbacks at News Media Organizations

Stem cell hype, as with science hyperbole more broadly, is a complex, multilayered phenomenon. We agree with science communication scholars who argue that it is a mistake to blame journalists as the dominant source of hyperbolic claims about science. Without wanting to scapegoat journalists, there have been important changes in the news media environment that pose significant challenges to in-depth critical news media coverage of scientific research.

Responsible news media coverage of scientific research benefits from scientists engaging with reporters skilled in such fields as science journalism and health reporting. Unfortunately, many talented and experienced science journalists and health reporters have experienced significant professional setbacks due to layoffs, staff reductions, the elimination of science and health journalism desks and beats, and the shuttering of outlets previously known for publishing high-quality science journalism and health reporting [15–17]. The loss of skilled science journalists and health reporters and the reduction in the number of news media platforms publishing serious science and health journalism could lead to concerning outcomes regarding how future stem cell science is communicated to the public. Without knowledgeable science journalists capable of evaluating work from their specialized fields critically before reporting, it may become easier for scientists, academic medical centers, and biotechnology companies to engage in hyperbole when using press releases and other promotional devices to draw attention to studies and research findings. Without sufficient time to report stories in depth and communicate with sources able to evaluate claims made by their scientific peers, journalists may struggle to perform in-depth science reporting. Absent the knowledge, critical assessment skills, and networks of sources acquired during years of working as a science journalist or health reporter, some journalists may be reduced to repackaging press releases. The use of “AI” tools to replace journalists risks accelerating this trend to repackage promotional material without adding depth, critical analysis, and meaningful context.

The long-term effects of staff layoffs at mainstream news media outlets and the shuttering of once prominent venues for science journalism and health reporting are unclear. However, the loss of skilled and experienced science and health reporters, editors, and producers could undermine the ability of news media platforms to provide high-quality news coverage and cover science-related stories in an accurate and informed manner. Such developments could be particularly consequential for news media coverage of stem cell research and regenerative medicine, where explaining highly technical and specialized studies in a publicly accessible manner requires the interpretive skills and effective communication styles of experienced science journalists. Previous research on science hype in general and stem cell hype in particular has paid insufficient consideration to the loss of news media outlets, the decline of jobs in journalism, and other dramatic changes taking place in the production of “news.”

## Promoting Responsible Science Communication with Guidelines and Codes of Practice is a Collective Responsibility between Scientists and Journalists

Scientists and journalists are crucial participants in the public communication of science. However, they operate within different organizational settings, have distinct workplace cultures and routines, and often must communicate with their anticipated audiences in very different ways. These differences prompt the question of what constitutes ethical and responsible science communication and whether there are any shared norms guiding the practices and professional activities of both journalists and scientists. Acknowledging the different organizational environments within which most journalists and scientists work, we suggest that one common value spanning these distinct workplace cultures is the basic norm of honesty, truthfulness, and accuracy in communication.

For stem cell scientists, to address rising concerns surrounding hyperbole and promote responsible scientific advances, the International Society for Stem Cell Research (ISSCR) developed and periodically revises detailed guidelines for the responsible communication of stem cell research and clinical translation [44]. In its guidelines, the ISSCR acknowledges that “inaccurate or incomplete representations can have tangible impacts on the expectations of the general public, patient communities, physicians, and on the setting of health and science policies” [44]. The guidelines note that such misrepresentations can be exploited by companies marketing unproven stem cell interventions. While the recommendations are intended specifically for stem cell researchers, the ISSCR guidelines contain valuable general advice concerning efforts to engage in responsible science communication. The guidelines provide practical insights that have the potential to inform how academic public affairs officers, communication and marketing specialists, and other contributors to science communication describe scientific studies and explain their significance.

Emphasizing the importance of making evidence-based, responsible, non-hyperbolic claims, the ISSCR guidelines provide three recommendations concerning public representation of science, communications about clinical trials, and communications about clinical care. Spanning the recommendations is a recurring call for balanced, honest, and accurate representation. For example, the ISSCR encourages awareness of how positive “spin” is sometimes applied to non-statistically significant results to justify follow-up findings and urges researchers to avoid premature reporting and rhetoric that might promote misunderstandings of investigational stem cell-based medical products by leading patients or clinical trial participants to construe them as established treatments. Particularly in the case of stem cell research, to avoid misleading members of the public, researchers and communications team members involved in framing public representations of scientific research need to be forthright about the rigorous nature of the translational process and the often-extended timelines required to test investigational medical products such as stem cell-based interventions and other regenerative medicine products. In addition, misrepresented research goals, findings, and applications should be corrected in a timely and accurate manner. The guidelines stress that forward-looking statements about potential research impacts should be “accurate, circumspect, and restrained” [44].

Unsurprisingly, existing guidelines developed by journalists concerned about promoting responsible news coverage also emphasize truth telling and identify such values such as accuracy, accountability, transparency, and integrity. For example, the Society of Professional Journalists urges its members to avoid misrepresentation and promptly correct inaccuracies [45], and the Association of Health Care Journalists calls for its members to “avoid vague, sensational language” and report both risks and benefits of any treatment [46]. These shared values span the fields of stem cell research and science journalism and health reporting, and provide insight into the core norms embedded in ethical, responsible science communication meant to promote public understanding.

Acknowledging the value of guidelines and formal standards of conduct, codes of practice must be part of broader efforts to eliminate stem cell hype and science hype in general. Scientists and journalists must hold themselves and each other accountable toward accountable science communication. Scientists themselves could play a more active role in promoting responsible science communication in their field. While some stem cell researchers have emphasized the importance of not engaging in hyperbole and using publicly engaged forms of science communication to “translate” research findings in an accessible manner, it is plausible that many scientists do not pursue science communication in this way because they view such activity as time-consuming and potentially damaging to their research careers [47, 48]. Such attitudes are connected to existing systemic pressures that contribute to a highly competitive academic research environment and assign little value to responsible science communication and public engagement. To nurture better public understanding trust of stem cell research and other scientific fields, it is imperative to transform such structural factors, create more spaces for public engagement, better reward researchers engaged in responsible science communication, and support accuracy and credibility in all efforts to communicate research findings and discuss the significance of particular fields of scientific research. We also support the recommendation that follow-up steps to these existing codes of practice should involve increased collaboration between scientists, journalists, and the public [49].

## Promoting Responsible Science Communication in a Social Media Era

Accurate representations of stem cell research and timely corrections of inaccurate claims or distorted results have become increasingly challenging in an era of rapid information spread enabled by online social media platforms [50–52]. Social media platforms provide valuable spaces for sharing scientific information and promoting public engagement not only because of their large and diverse user bases, but also due to the interactive nature of these platforms. This important feature enables direct communication between scientists, institutions, journalists, and members of the public [12–14, 53–55]. While social media platforms are used by scientists, academic institutions and other research facilities, and reputable news media outlets, they are of course also used by many other parties. And though social media platforms can be used to distribute valuable information about stem cell research, they can also promote the circulation of science- and health-related misinformation and disinformation [56]. For example, numerous clinics marketing unapproved stem cell therapies use X/Twitter and leverage social media influencers to advertise their products to the general public [56, 57]. A study conducted on X/Twitter found that fake news travels substantially faster than accurate stories. Furthermore, circulation of such misinformation is due to the activities of humans rather than online bots [58]. Social media platforms are important sites for engaging in science communication. However, they also enable the circulation and amplification of hyperbolic claims, efforts to “spin” findings from particular studies, and other misrepresentations.

To sustain responsible science communication in the social media era, stem cell scientists and their institutions need to develop new strategies to support accurate representations of scientific research on social media platforms. According to Alan Regenberg, social media currently offers “a useful adjunct, but not a plausible substitute for existing structures for reviewing, approving, and communicating” science [54]. Acknowledging the continuing role of funding agencies, peer-reviewed journals, and pre-print servers as sites for reviewing and evaluating scientific studies and research findings, social media platforms are increasingly important as sites for communicating scientific research beyond professional contexts to wider publics. However, it is also critical that scientists hold each other accountable toward communicating their science with accuracy.

The strategies currently used to detect, flag, and constrain misrepresentations of science across social media platforms are imperfect. Fact-checkers often are not experts in particular scientific fields, and AI algorithms rely on the quality of data used to train them and are less able than human reviewers to understand nuance and context [59]. Given the speed at which information can spread on social media, it is not possible to fact-check every social media post in a timely manner. As a result, misrepresentations can easily proliferate. This phenomenon is particularly evident in the social media streams of clinics using such platforms to market unproven and unapproved interventions [56, 57, 60]. A multilayered approach to fact-checking which utilizes collective efforts from human experts, crowdsourcing, and AI will likely be the most effective strategy for supporting responsible science communication on social media [61]. Furthermore, disincentivizing the production and circulation of disinformation and misinformation might be a promising strategy to address the spread of misrepresentations of science on social media platforms.

Nielsen offers other recommendations intended to manage misinformation and disinformation while protecting freedom of expression [62]. In cases where disinformation can cause tangible harm, Nielsen stresses the importance of proportionate legal responses. Such measures should be carefully calibrated. Another suggestion involves platform-level interventions such as flagging disinformation or removal of user content, though platform responses should reflect alignment between platform policy and international human rights principles such as due process. Nielsen emphasizes the need for oversight mechanisms that ensure platform decisions are accountable and open to scrutiny. Such mechanisms include transparency about content moderation policies, independent audits, and opportunities for users to appeal content moderation decisions [62]. Collectively, these recommendations support a framework for countering scientific misinformation and disinformation in digital spaces while also respecting civil liberties.

With X/Twitter becoming increasingly polarized following the cutdown of content moderation as well as additional fees to access further platform privileges, many scientists have turned to other social media platforms including former X/Twitter project and free, open-source social media platform Bluesky [63–67]. While scientists have identified more content moderation on this platform as a key promising factor in their migration from X/Twitter, it remains to be seen whether Bluesky and other social media platforms will be long-lasting community spaces for supporting responsible science communication.

## Conclusion

Responsible, evidence-based science communication plays a crucial role in promoting public understanding of science and technology and helping citizens understand and address the ethical, legal, and social implications of scientific research. However, it is important to understand the many barriers to promoting meaningful science communication and the difficulties associated with supporting accurate representations of scientific research in news media coverage, on social media platforms, and elsewhere. Structural changes in the incentive and disincentive structures that shape contemporary scientific research are needed to foster organizational cultures and research environments that value and reward honesty and transparency in research. Developing and enforcing guidelines for communicating scientific research in an ethical, responsible manner represents one important approach for articulating standards and providing practical recommendations for evidence-based communication. In a technological era of digital communications, social media platforms have an important role to play in promoting standards for science communication and ensuring that online communication spaces do not contribute to the circulation of health- and science-related misinformation and disinformation. Finally, academic institutions and other research organizations should better value and reward responsible science communication. New incentive structures for researchers and efforts to build more collaborative institutional cultures have the potential to play an important role in supporting approaches to research that emphasize accuracy and trustworthiness over hyperbole.

## Data Availability

No datasets were generated or analysed during the current study.
